# Comparison between 1,2-Dihydropyridine and 1,4-Dihydropyridine on Hydride-Donating Ability and Activity

**DOI:** 10.3390/molecules27175382

**Published:** 2022-08-24

**Authors:** Jin-Ye Zhang, Xiao-Qing Zhu

**Affiliations:** The State Key Laboratory of Elemento-Organic Chemistry, College of Chemistry, and Collaborative Innovation Center of Chemical Science and Engineering, Nankai University, Tianjin 300071, China

**Keywords:** 1,2-dihydropyridine, 1,4-dihydropyridine, thermodynamic, kinetic, thermo-kinetic parameters

## Abstract

In this paper, detailed comparisons of the driving force in thermodynamics and intrinsic force in the kinetics of 1,2-dihydropyridine and 1,4-dihydropyridine isomers of PNAH, HEH, and PYH in hydride transfer reactions are made. For 1,2-PNAH and 1,4-PNAH, the values of the thermodynamic driving forces, kinetic intrinsic barriers, and thermo-kinetic parameters are 60.50 and 61.90 kcal/mol, 27.92 and 26.34 kcal/mol, and 44.21 and 44.12 kcal/mol, respectively. For 1,2-HEH and 1,4-HEH, the values of the thermodynamic driving forces, kinetic intrinsic barriers, and thermo-kinetic parameters are 63.40 and 65.00 kcal/mol, 31.68 and 34.96 kcal/mol, and 47.54 and 49.98 kcal/mol, respectively. For 1,2-PYH and 1,4-PYH, the order of thermodynamic driving forces, kinetic intrinsic barriers, and thermo-kinetic parameters are 69.90 and 72.60 kcal/mol, 33.06 and 25.74 kcal/mol, and 51.48 and 49.17 kcal/mol, respectively. It is not difficult to find that thermodynamically favorable structures are not necessarily kinetically favorable. In addition, according to the analysis of thermo-kinetic parameters, 1,4-PNAH, 1,2-HEH, and 1,4-PYH have a strong hydride-donating ability in actual chemical reactions.

## 1. Introduction

Dihydropyridine (DHP) is the active structural core of a wide variety of natural products, drugs, and functional materials [1]. Of the five possible isomers, only 1,2 and 1,4-DHP have been studied in depth [2,3,4,5,6,7,8,9,10,11,12,13,14,15,16]. Among them, 1,4-DHP is closest to NAD(P)H coenzyme; its biological applications are particularly extensive [17]. The active center of many drugs, such as nifedipine and amlodipine, is 1,4-DHP. Because it contains a chiral center, 1,2-DHP is mostly used as an important raw material for the active skeleton of natural alkaloids such as ibogaine, dioscorine, and the antiviral drug oseltamivir phosphate [18,19]. As organic hydride ion donors, both 1,2-DHP isomer and 1,4-DHP isomer have the same one-step hydride transfer mechanism when reacting with some negative ions, such as acridine perchlorate, 4-acetamido-2,2,6,6-tetramethyl-1-oxopiperidinium perchlorate, and 9-phenyl-2,3-dihydroxanthylium perchlorate (AcrH^+^ClO_4_^−^, Tempo^+^ClO_4_^−^ and PhXn^+^ClO_4_^−^) [20,21,22,23,24]. An interesting question arises here: what is the difference between 1,2-DHP and 1,4-DHP in their hydride-donating ability? 

**Scheme 1:**
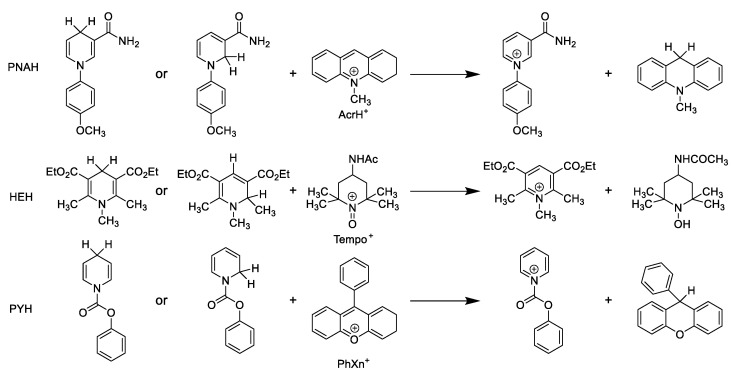
1,2-DHP and 1,4-DHP isomers hydride transfer reaction equation.

To answer the above question, we chose three types of usual manmade NADH analogues, phenyl-1,4-dihydronicotinamide (1,4-PNAH) and phenyl-1,2-dihydronicotinamide (1,2-PNAH), N-CH_3_-1,2-Hantzsch (1,2-HEH) and N-CH_3_-1,4 Hantzsch (1,4-HEH), and 1-phenoxyacyl-1,2-dihydropyridine (1,2-PYH) and 1-phenoxyacyl-1,4-dihydropyridine (1,4-PYH), as the research objects (Scheme 1). Furthermore, in this work, bond dissociation free energy [ΔG°(XH)] as the thermodynamic driving force was used to discuss the hydride-donating ability of the above NADH analogues in terms of thermodynamics [25]. It is well-known that the thermodynamic driving force for the self-exchange transfer reaction of hydride ions is 0 kcal/mol (XH^−^ + X^+^→X^+^ + XH^−^). Therefore, the activation free energy of the self-exchange reaction (ΔG^≠^_XH/X_) is used to describe the kinetic intrinsic barrier of the hydride donor. This indicates the kinetic intrinsic barrier of the compound itself in the chemical reaction. Thermo-kinetic parameters [ΔG^≠^°(XH)] are used to describe the actual hydride-donating ability of NADH analogues in chemical reactions [26]. It should be noted that in previous research reports of our group, we combined thermodynamic parameter [ΔG°(XH)] and kinetic parameter (ΔG^≠^_XH/X_) to define a new compound’s intrinsic physical parameter, which was named the thermo-kinetic parameter [ΔG^≠^°(XH)] [27]. According to the definition of the thermo-kinetic parameter (Equation (3)), the ΔG^≠^°(XH) value is determined by the value of ΔG°(XH) and the value of ΔG^≠^_XH/X_, and its value reflects the actual hydride-donating ability of the compound in a hydride transfer reaction. The larger the ΔG^≠^°(XH) value, the weaker the hydride-donating ability, and the smaller the ΔG^≠^°(XH) value, the stronger the hydride-donating ability.

## 2. Results

**Figure 1:**
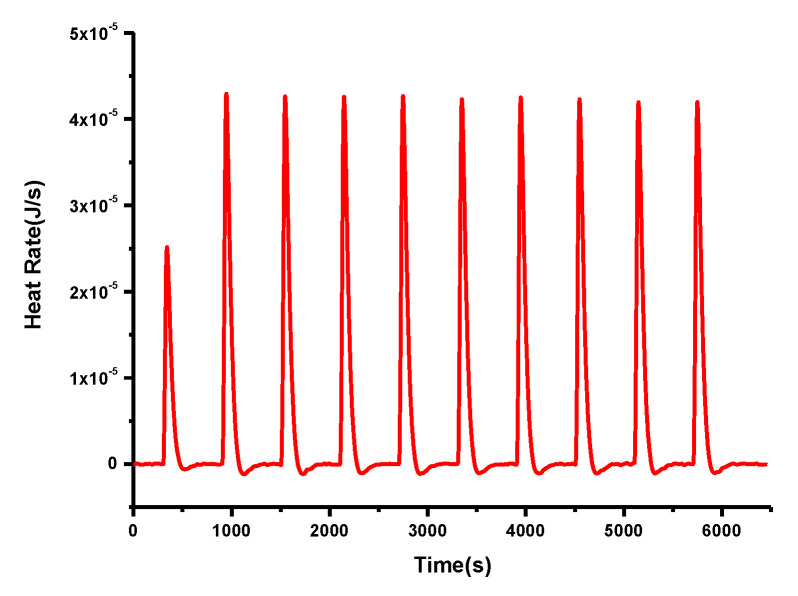
Isothermal titration calorimetry (ITC) for the reaction heat of 1,2−PYH with PhXn^+^ in CH_3_CN at 298 K. Titration was conducted by adding 10 μL of 1,2−PYH (2.34 mM) every 600 s into the CH_3_CN containing PhXn^+^ (ca.10 mM).

**Figure 2:**
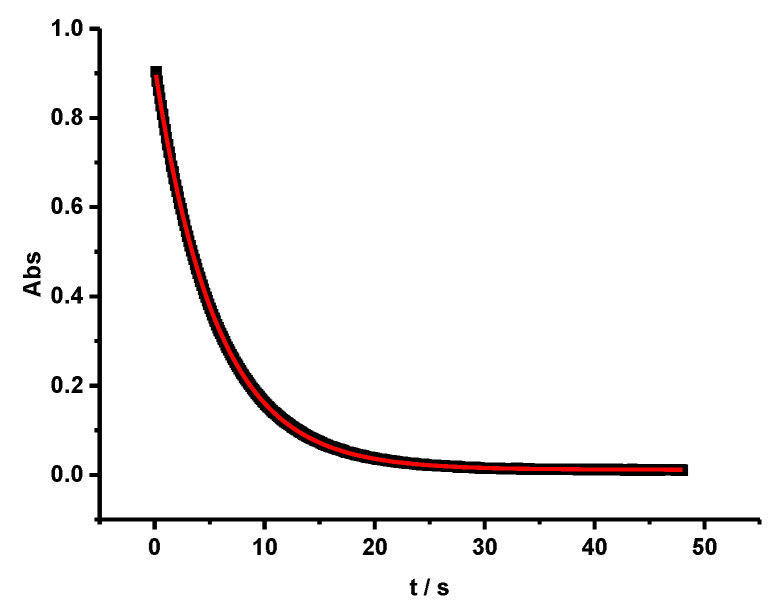
Time profile of the UV absorbance at 372 nm due to PhXn^+^ for the reactions of PhXn^+^ (0.1 mM) with 1,2-PYH (2 mM) in CH_3_CN at 298 K.

Two dihydrogen isomers of PNAH, HEH, and PYH were synthesized according to the literature methods and were identified by 1H NMR; the detailed data are listed in the Supporting Information [20,22,28,29,30,31]. The enthalpy change of the two dihydrogen isomers reacting with hydride acceptors was determined in acetonitrile using an isothermal titration calorimeter (CSC-4200 ITC) at 298 K as described previously (Figure 1) [32]. All kinetic tests were monitored in 298 K dry and anaerobic acetonitrile using an Applied Photophysics SX.18MV-R stopped-flow apparatus (Figure 2). The second rate constant (*k_2_*), activation free energies (ΔG^≠^_XH/Y_), and molar free energies ΔG°(XH/Y) of the three group reactions are listed in Table 1; see also Scheme 1. According to the data in Table 1; Table 2; and Equations (1)–(3), the thermodynamic driving forces, self-exchange reaction activation energies, and thermo-kinetic parameters values of 1,2/4-PNAH, 1,2/4-HEH, and 1,2/4-PYH are easily obtained (Table 3).

ΔG° = ΔG°_H-D_(XH) + ΔG°_H-A_(Y^+^) (1)

ΔG^≠^_XH/Y_ = ΔG^≠^°(XH) + ΔG^≠^°(Y^+^) (2)

ΔG^≠^°(XH) = 1/2[ΔG^≠^_XH/X_ + ΔG°(XH)] (3)

## 3. Discussion

### 3.1. Analysis of Thermodynamic Driving Forces of 1,2/4-PNAH, 1,2/4-HEH, and 1,2/4-PYH as Hydride Donors in Acetonitrile at 298 K

As shown in Table 3, obviously, the heterolytic bond dissociation free energies of 1,2-PNAH and 1,4-PNAH, 1,2-HEH and 1,4-HEH, and 1,2-PYH and 1,4-PYH were 60.50 and 61.90 kcal/mol, 63.40 and 65.00 kcal/mol, and 69.90 and 72.60 kcal/mol, respectively. Whether it is 1,2-DHP or 1,4-DHP isomers, the order of ΔG°(XH) is PYH > HEH > PNAH. This indicates that PNAH has the best hydride-donating ability in thermodynamics, and the positive ion salt of PYH is commonly used as a hydride ion acceptor. In addition, the ΔG°(XH) of all the 1,4-DHP isomers was larger than that of the 1,2-DHP isomers, which indicates that the 1,2-DHP isomers have a better hydride-donating ability in thermodynamics. 

### 3.2. Analysis of Kinetic Intrinsic Barriers of 1,2/4-PNAH, 1,2/4-HEH, and 1,2/4-PYH as Hydride Donors in Acetonitrile at 298 K

As shown in Table 3, the ΔG^≠^_XH/X_ values of 1,2-PNAH and 1,4-PNAH, 1,2-HEH and N-1,4-HEH, and 1,2-PYH and 1,4-PYH were 27.92 and 26.34 kcal/mol, 31.68 and 34.96 kcal/mol, and 33.06 and 25.74 kcal/mol, respectively. For 1,2-DHP isomers, the order of ΔG^≠^_XH/X_ is 1,2-PYH > 1,2-HEH > 1,2-PNAH. It is shown that 1,2-PNAH has the best hydride-donating ability, then 1,2-HEH, and 1,2-PYH has the worst hydride-donating ability in dynamics. However, for the 1,4-DHP isomers, the order of hydride-donating ability changed. The order of ΔG^≠^_XH/X_ was 1,4-HEH > 1,4-PNAH > 1,4-PYH, which indicates that the alteration of the structure has a great effect on ΔG^≠^_XH/X_. The rule of the ΔG^≠^_XH/X_ of the three groups of isomers was also different. The order of ΔG^≠^_XH/X_ for the three groups of isomers was 1,2-PNAH > 1,4-PNAH, 1,2-HEH < 1,4-HEH, and 1,2-PYH > 1,4-PYH, respectively, which indicates that the effect of structure on ΔG^≠^_XH/X_ is not a single rule. In addition, this also shows that the laws of the hydride-donating ability of three group dihydrogen isomers in kinetics and thermodynamics are almost completely different. Therefore, it is unscientific to use a single thermodynamic or kinetic parameter to analyze the hydride-donating ability of a compound.

### 3.3. Analysis of Thermo-Kinetic Parameters of 1,2/4-PNAH, 1,2/4-HEH, and 1,2/4-PYH as Hydride Donors in Acetonitrile at 298 K

As shown in Table 3, the ΔG^≠^°(XH) values of 1,2-PNAH and 1,4-PNAH, 1,2-HEH and 1,4-HEH, and 1,2-PYH and 1,4-PYH were 44.21 and 44.12 kcal/mol, 47.54 and 49.98 kcal/mol, and 51.48 and 49.17 kcal/mol, respectively. For 1,2-DHP isomers, the order of Δ*G*^≠^°(XH) was 1,2-PYH > 1,2-HEH > 1,2-PNAH. This indicates that 1,2-PNAH is the best hydride donor in actual hydride transfer reactions, then 1,2-HEH, and 1,2-PYH is the worst hydride donor in actual hydride transfer reactions. Additionally, for 1,4-DHP isomers, the order of ΔG^≠^°(XH) is 1,4-HEH > 1,4-PYH > 1,4-PNAH, which indicates that 1,4-PNAH is the best hydride donor in chemical reactions, then 1,4-PYH, and 1,4-HEH is the worst hydride donor in chemical reactions. 

Furthermore, the order of ΔG^≠^°(XH) for the three groups of isomers is 1,2-PNAH > 1,4-PNAH, 1,2-HEH < 1,4-HEH and 1,2-PYH > 1,4-PYH, respectively. For PNAH, the thermodynamic driving force of 1,4-PNAH was 1.4 kcal/mol larger than that of 1,2-PNAH, and the kinetic intrinsic barrier was 1.58 kcal/mol smaller than that of 1,2-PNAH. Therefore, the difference in thermodynamic driving force shows that 1,4-PNAH has a smaller thermo-kinetic parameter, which indicates that 1,4-PNAH is a better hydride donor. For HEH, the thermodynamic driving force of 1,4-HEH was 1.6 kcal/mol larger than that of 1,2-HEH, and the kinetic intrinsic barrier was 3.28 kcal/mol larger than that of 1,2-HEH, which indicates that the difference in the thermodynamic driving force and the difference in the kinetic intrinsic barrier together mean that 1,2-HEH has a smaller thermo-kinetic parameter. This also means that 1,2-HEH has a better hydride-donating ability. Additionally, for PYH, the thermodynamic driving force of 1,4-PYH was 2.7 kcal/mol larger than that of 1,2-PYH, and the kinetic intrinsic barrier was 7.32 kcal/mol smaller than that of 1,2-PYH. This shows that the difference in the kinetic intrinsic barrier means that 1,4-PYH has a smaller thermo-kinetic parameter and a better hydride-donating ability. The internal reasons affecting the order of the thermo-kinetic parameters of the above three groups of compounds are dominated by the kinetic intrinsic barrier, co-dominated by a kinetic intrinsic barrier and thermodynamic driving force, and dominated by the thermodynamic driving force, respectively. In addition, it also shows that there is no linear relationship between the active site and the thermo-kinetic parameter of the dihydrogen isomers, and it is not advisable to infer a hydride-donating ability based on the structure of dihydrogen isomers. It is not difficult to find that the above analysis results are different from the results of the thermodynamic analysis and kinetic intrinsic barrier analysis. Therefore, the method of judging the hydride-donating ability only by the thermodynamic analysis and kinetic intrinsic barrier analysis is wrong.

## 4. Conclusions

In this work, we compared the hydride-donating ability of three groups of 1,2-DHP and 1,4-DHP dihydrogen isomers using thermodynamic parameters, kinetic parameters, and thermo-kinetic parameters. When describing the hydride-donating ability of NADH analogues, it is necessary to combine kinetic and thermodynamic parameters into thermo-kinetic parameter analysis instead of using a single experimental result. The order of the actual hydride-donating ability of the 1,2-DHP isomers and the 1,4-DHP isomers described by thermo-kinetic parameters is 1,2-PNAH > 1,2-HEH > 1,2-PYH and 1,4-PNAH > 1,4-PYH > 1,4-HEH, respectively. For the three groups of dihydrogen isomers, the actual hydride-donating ability described by thermo-kinetic parameters is 1,4-PNAH > 1,2-PNAH, 1,2-HEH > 1,4-HEH, and 1,4-PYH > 1,2-PYH, respectively. This indicates that there is no fixed linear relationship between the isomers’ structure and the actual hydride-donating ability. This method is not only applicable when describing hydride-donating abilities but also in describing hydrogen-donating abilities, proton-donating abilities, electron-donating abilities, etc.

## Figures and Tables

**Table 1:** Second-order rate constants (*k*_2_), activation free energies (ΔG^≠^), and molar free energy change (ΔG°) of oxidations of dihydropyridine compounds in CH_3_CN at 298 K.

	PNAH + AcrH^+^		HEH + TEMPO^+^		PYH + PhXn^+^	
	1,2-PNAH	1,4-PNAH	1,2-HEH	1,4-HEH	1,2-PYH	1,4-PYH
*k*_2_ *^a^*	10.06	12.30	1.44 × 10^5^	2.34 × 10^3^	9.50 × 10^−1^	4.76 × 10^1^
ΔG^≠ *b*^	16.05	15.96	10.41	12.85	17.47	15.16
ΔG° *^c^*	−15.70	−14.30	−37.30	−35.70	−21.70	−19.00

*^a^ k*_2_ (M^−1^ s^−1^) is the second-order rate constant of the hydride transfer in CH_3_CN at 298 K. The uncertainty is smaller than 5%. *^b^* Derived from Eyring equation (T = 298 K), the unit is kcal/mol. *^c^* ΔG° is equal to the corresponding reaction heat; the latter was measured by titration calorimetry in CH_3_CN at 298 K. The data given in kcal/mol are the average values of at least three independent runs.

**Table 2:** Bond formation free energy and thermo-kinetic parameters of hydride acceptor used in this work (T = 298 K).

Compounds	ΔG°(Y^+^) *^a^*	ΔG^≠o^(Y^+^) *^b^*
AcrH+	−76.2	−28.16
TEMPO+	−100.7	−37.13
PhXn+	−91.6	−34.01

*^a^*^,*b*^ Reference from [30]; the unit is kcal/mol.

**Table 3:** Thermodynamic driving forces [ΔG°(XH)], self-exchange reaction activation energies [ΔG^≠^_XH/X_], and thermo-kinetic parameters [ΔG^≠^°(XH)] of 1,2/4-PNAH, 1,2/4-HEH, and 1,2/4-PYH as hydride donors at 298 K in CH_3_CN at 298 K.

	1,2-PNAH	1,4-PNAH	1,2-HEH	1,4-HEH	1.2-PYH	1,4-PYH
ΔG°(XH)	60.50	61.90	63.40	65.00	69.90	72.60
ΔG^≠^_XH/X_	27.92	26.34	31.68	34.96	33.06	25.74
ΔG^≠^°(XH)	44.21	44.12	47.54	49.98	51.48	49.17

The unit is kcal/mol.

## Data Availability

Not applicable.

## Supplementary Materials

The following supporting information can be downloaded at: https://www.mdpi.com/article/10.3390/molecules27175382/s1. The detailed synthesis method of the compound are shown in SI. Detailed 1H NMR and 13C NMR data of typical compounds are shown in SII [20,22,28,29,30,31].

## Sample Availability

Samples of the compounds are available from the authors.
