# Supplementary material for: Comparison between 1,2-Dihydropyridine and 1,4-Dihydropyridine on Hydride-Donating Ability and Activity
Source: Molecules. 2022 Aug 24;27(17):5382. doi: 10.3390/molecules27175382 (PMC9457676; doi:10.3390/molecules27175382)
Supplement: Supplementary file 1 [file molecules-27-05382-s001.zip › molecules-1851563-supplementary.pdf]

Supporting Information

Comparison between 1,2-Dihydropyridine and 1,4-Dihydropyridine on Hydride-Donating Ability and Activity

Jin-Ye Zhang*, and Xiao-Qing Zhu

The State Key Laboratory of Elemento-Organic Chemistry, Department of Chemistry, Collaborative Innovation Center of Chemical Science and Engineering, Nankai University, Tianjin 300071, China

*Corresponding author:

Dr. Jin-Ye Zhang, The State Key Laboratory of Elemento-Organic Chemistry, Department of Chemistry, Collaborative Innovation Center of Chemical Science and Engineering, Nankai University, Tianjin 300071, China. E-mail: zjynku@163.com

Content

		Page
SI.	^{1}H NMR of the Representative Compounds	S2-7
SII.	^{1}H NMR of the Representative Compounds	S7-12

SI. ^{1}H NMR of the typical Compounds

Syntheses of AcrH$^+$ClO$_4^-$:[1]

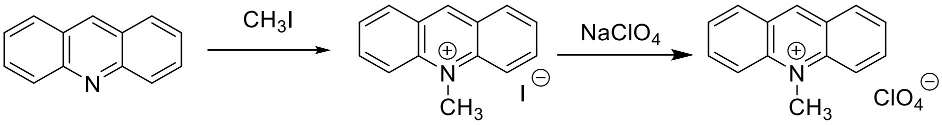

The acridine (10 mmol) and CH_3I (30 mmol) were added in CH_3CN (50 ml), the mixture was refluxed for 7 h. After the solution cooled to room temperature, the product was precipitated and filtered to give AcrH$^+$I$^-$. 1 mmol of AcrH$^+$I$^-$ was dissolved in supersaturated aqueous solution of $NaClO_4$. Orange solid precipitated soon after heating to reflux. AcrH$^+$ClO$_4^-$ was obtained after 2 recrystallizations from ethanol.

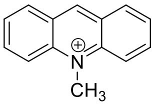

^{1}H NMR(DMSO-*d*$_6$, 400MHz): 4.87(s, 3H), 8.06(t, 2H), 8.48(m, 2H), 8.64(dd, 2H), 8.80(d, 2H), 10.20(s, 1H)

Syntheses of PhXn$^+$ ClO$_4^-$:[2]

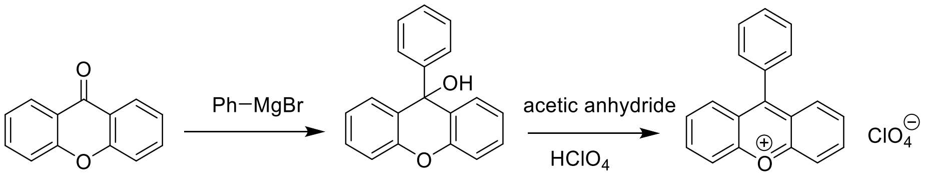

Xantone (8 mmol) was dissolved in 50 ml THF, the Ph-MgBr Grignard reagent (16 mmol) was added in the mixture in the protection of N_2. The resulted mixture was stirred at room temperature for 2 h, then the $NaHCO_3$ aqueous solution was added to the mixture, the residue was extracted with CH_2Cl_2, and dried over Na_2SO_4, concentrated then dried to give crude product. The crude product was used for the following reactions without further purification. The crude product (5 mmol) was dissolved in acetic anhydride, $HClO_4$ (70 %, 4ml) was added in the mixture at 0 ^{o}C. Excessive Et_2O was added to give PhXn$^+$ClO$_4^-$ as precipitation, filtered and washed with Et_2O to give PhXn$^+$ClO$_4^-$, yield 75%.

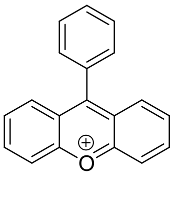

^{1}H NMR (CD_3CN, 400M MHz): 7.44 (d, 2H); 7.78 (d, 2H); 8.02 (t, 2H); 8.38 (m, 5H); 8.60 (t, 2H);

Syntheses of TEMPO$^+$ClO$_4^-$:[3]

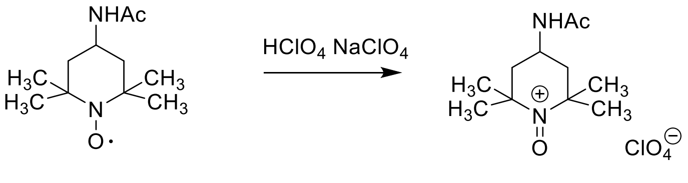

In a 500ml three-necked bottle, add 50g of TEMPO radicals and 100ml of water, and stir evenly. The

solution prepared by 33g of 70% concentration of perchloric acid aqueous solution and 25mL of water was slowly added dropwise into the three-necked bottle. Subsequently, 181.5 g of a 5.25% strength NaClO aqueous solution was slowly added dropwise. After cooling in an ice bath, suction filtration. Wash with ice-cold 100 ml of $NaHCO_3$ aqueous solution, 100 ml of water and CH_2Cl_2 successively. The solid was recrystallized from water and dried in vacuo, yield 75%.

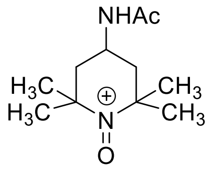

^{1}H NMR (CD_3CN, 400M MHz): 1.56 (s, 6H), 1.90 (s, 6H), 2.48 (s, 3H), 2.68 (t, 2H), 2.82 (m, 2H), 5.22 (m, 1H), 9.36 (s, 1H).

Syntheses of PNAH:[4]

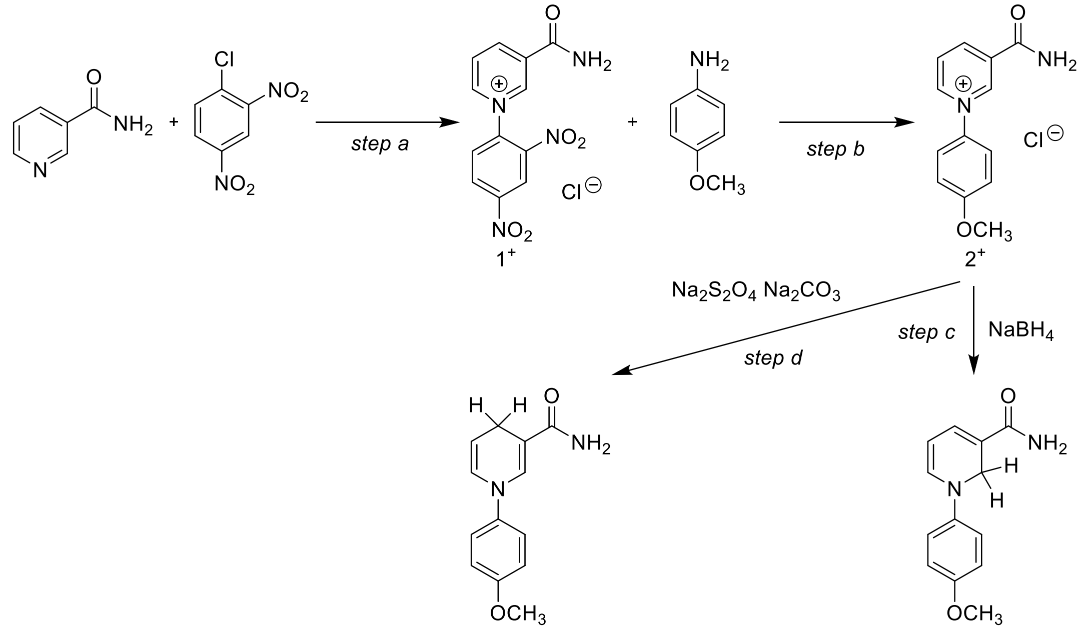

Step a:

Nicotinamide (10 g, 0.082 mol) and 2,4-dinitrochlorobenzene (40 g 1.98 mol) were placed in a 250 ml round bottom flask. The product 1^+ was obtained after heating at 100 °C for 1 h. The product was extracted with water and ether, and the water layer was spin-dried to obtain the product 1^+.

Step b:

An equivalent amount of 1^+ and p-methoxyaniline was added to an appropriate amount of methanol, heated to reflux until the color of the system turned yellow. Cool the reaction, remove the solvent by rotary evaporation, extract with water and ethyl acetate, take the water layer and spin dry to obtain the product 2^+.

Step c:

Under argon, 6 mmol of 2^+ was placed in a 50 ml Slake bottle, 2 mL of 2N NaOH aqueous solution was added, and then 5 ml of methanol was added, dissolved by ultrasonic wave, and cooled to 0 °C. 0.24g of $NaBH_4$ was added in batches, and the reaction was continued for 30min after the addition. Filtration gave a yellow precipitate, which was washed with water and dried. The product was purified using column chromatography

to give the product 1,2-PANH.

Step d:

0.096 mol $Na_2S_2O_4$ and 0.052 mol Na_2CO_3 were added in 40 ml H_2O, the aqueous solution (mark as solution A) was removed oxygen by bubbling with pure argon gas for 15 min. 0.012 mol 3^+ was dissolved in 20 ml H_2O (mark as solution B), and solution B was removed oxygen by bubbling with pure argon gas for 15 min. The solution B was slowly dropped into solution A within 30 min, the mixture was stirred for 4 h at room temperature to get crude product 1,4-PNAH. The crude was recrystallized from mixed solvent (H_2O and ethanol) to give pure 1,4-PNAH (yield 40%).

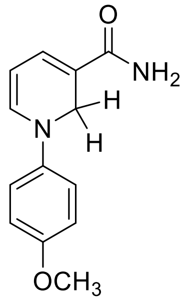

^{1}H NMR (400 MHz, DMSO) 3.73 (s, 3H), 4.41 (s, 2H), 5.10 (t, 1H), 6.76 – 7.01 (m, 6H), 7.02 – 7.09 (m, 2H).
^{13}C NMR (101 MHz, DMSO) δ 46.46, 55.77, 98.92, 114.78, 114.95, 115.21, 117.49, 118.09, 128.90, 136.81, 138.81, 155.28, 167.78.

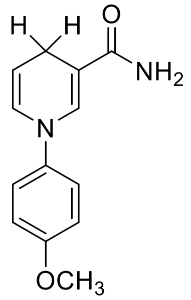

^{1}H NMR (400 MHz, $CDCl_3$) δ 3.42 (dt, 2H), 3.99 (d, 3H), 5.10 (ddd, 1H), 5.69 (s, 2H), 6.41 (dt, 1H), 6.99 – 7.10 (m, 2H), 7.25 (dd, 2H), 7.62 (d,1H).
^{13}C NMR (101 MHz, $CDCl_3$) 22.88, 55.55, 101.26, 104.00, 114.67, 121.24, 128.29, 137.44, 137.62, 156.85, 170.13.

Syntheses of HEH: [5]

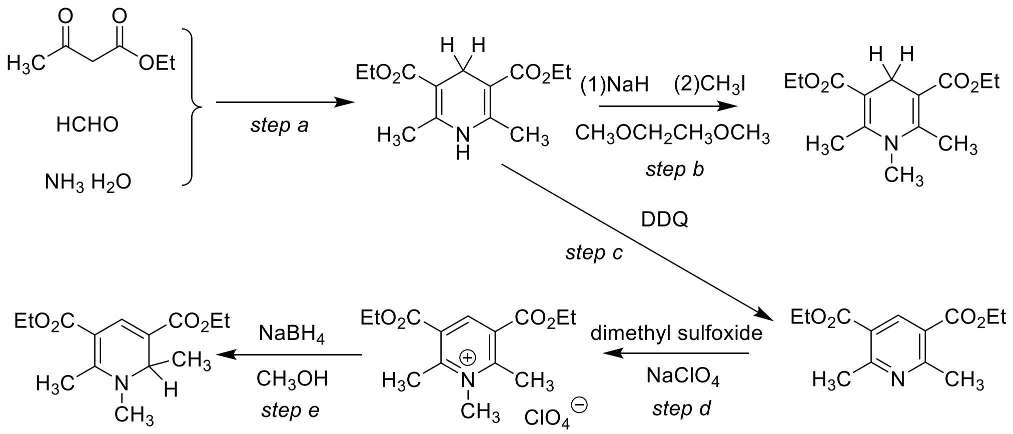

Step a:

0.12 mol ethyl acetoacetate, 80 ml methanol, 0.06 mol formaldehyde and 0.06 mol $NH_3 \bullet H_2O$ (25%) were added in 250 ml round bottom flask. The solution was refluxed for 8 h, and then the solution was cooled to room temperature. The precipitation was filtered and recrystallized from methanol to give HEH (N-H).

Step b:

Add 35 mL of dry 1,2-dimethoxyethane to a 100 mL three-necked flask, and dissolve 2.5 mmol of N-H HEH in it. The solution was passed through argon for 30 minutes to remove the oxygen in the system, and 0.72 g of NaH was added under an argon-protected ice-water bath, and a large amount of gas was generated. After the gas was no longer released, 2 mL of CH_3I was added under the ice-water bath. After the addition, the ice-water bath was removed, and the reaction was continued at room temperature for 4 hours. Under the protection of argon in an ice-water bath, a large amount of water was slowly added dropwise, and a solid was precipitated. Suction filtration, vacuum drying, and recrystallization from methanol to obtain 1,4-HEH (N-CH_3). The yield is 80%.

Step c:

In a 250 mL round-bottomed flask, add 10 mmol HEH (N-H), 100 mL dichloromethane, add 10 mmol DDQ in batches with stirring, filter, and spin dry the filtrate. Using a mixed solvent of petroleum ether and ethyl acetate as an eluent to pass through a silica gel column to obtain dehydrohans Hantzsch.

Step d:

In a 100 mL round-bottom flask, add 5 mmol of dehydrohans Hantzsch, 2 mL of dimethyl sulfate, and stir at 70℃ for 6 hours. After cooling, saturated aqueous $NaClO_4$ solution was added, and the mixture was stirred overnight, and a white solid was precipitated. Suction filtration, wash with water and diethyl ether in turn, recrystallize with methanol, suction filtration, and vacuum dry to obtain Hantzsch perchlorate.

Step e:

In a 250 mL three-necked flask, add 3 mmol of Hantzsch perchlorate, add 100 mL of anhydrous methanol, degas with argon for 30 minutes, and at room temperature, add $NaBH_4$ in batches until the reaction solution becomes a transparent yellow solution . An aqueous solution containing 2 g of sodium bicarbonate was added to quench the reaction, filtered, and the methanol in the system was removed by rotary evaporation, extracted with dichloromethane, and dried over anhydrous sodium sulfate. Filter, spin dry, use petroleum ether and ethyl acetate as eluent, and pass through silica gel column to obtain 1,2-HEH (N-CH_3) as yellow powder. The yield is 55%.

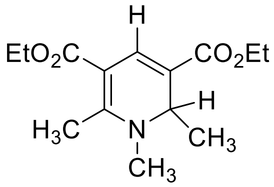

^{1}H NMR (400 MHz, $CDCl_3$) 1.01 (dd, 3H), 1.19 (ddt, 6H), 2.41 (d, 3H), 3.09 (d, 3H), 4.08 (dqd, 4H), 4.40 (q, 1H), 7.59 (s, 1H).
^{13}C NMR (101 MHz, $CDCl_3$) δ 14.41, 14.51, 16.67, 16.92, 38.53, 56.93, 59.20, 59.81, 97.81, 109.43, 134.53, 159.87, 165.74, 166.70.

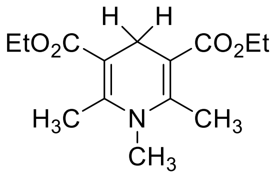

^{1}H NMR (400 MHz, $CDCl_3$) δ 1.26 (td, 6H), 2.35 (s, 6H), 3.12 (s, 5H), 4.14 (qd, 4H).
13C NMR (101 MHz, CDCl3) δ 14.41, 15.93, 23.95, 33.82, 59.77, 101.53, 150.59, 168.04.

Syntheses of PYH:[6]

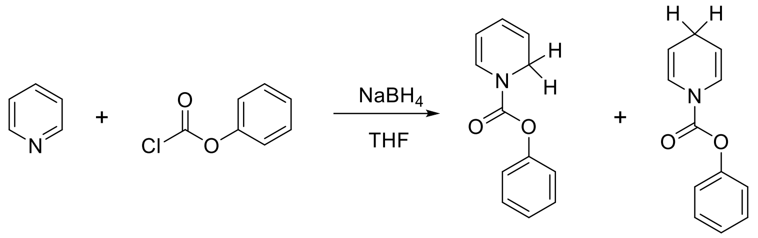

Step a：

Equivalents of pyridine and $NaBH_4$ were added to 120 ml of THF under argon. After the system was cooled to -10°C, an equivalent amount of Phenyl chloroformate was slowly added dropwise. After 5 h of reaction, a large amount of water was added to quench the reaction. Extract with ether, dry over anhydrous sodium sulfate, and spin dry. 1,2-PYH and 1,4-PYH can be obtained by passing through the chromatographic column. Yields were 20% and 10%, respectively.

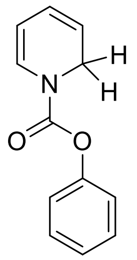

1H NMR (400 MHz, $CDCl_3$) 4.26 – 4.36 (m, 1H), 4.38 – 4.53 (m, 1H), 5.13 (dt, 1H), 5.45 (tt, 1H), 5.69 – 5.83 (m, 1H), 6.71 (dd, 1H), 7.00 (d, 2H), 7.10 (q, 1H), 7.24 (t, 2H).
^{13}C NMR (101 MHz, $CDCl_3$) 43.84, 44.32, 105.88, 106.08, 118.96, 119.53, 121.61, 121.89, 122.30, 125.42, 125.73, 125.77, 126.13, 129.42, 151.00, 151.58.

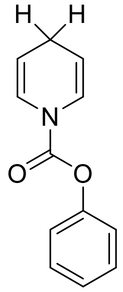

^{1}H NMR (400 MHz, $CDCl_3$) 2.84 (dq, 2H), 4.83 – 4.95 (m, 1H), 4.95 – 5.05 (m, 1H), 6.78 (d, 1H), 6.84 (d, 1H), 7.09 (d, 2H), 7.32 – 7.38 (m, 3H).
^{13}C NMR (101 MHz, $CDCl_3$) δ 22.49, 106.78, 107.40, 121.58, 123.43, 123.60, 125.84, 126.32, 129.59, 149.97, 150.77.

References

1. Shen, G.B., et al., *Prediction of Kinetic Isotope Effects for Various Hydride Transfer Reactions Using a New Kinetic Model.* J Phys Chem A, 2016. **120**(11): p. 1779-99.
2. Xiao-Qing Zhu, Y.T., and Chao-Tun Cao, *Thermodynamic Diagnosis of the Properties and Mechanism of Dihydropyridine-Type Compounds as Hydride Source in Acetonitrile with "Molecule ID Card".* J. Phys. Chem. B, 2010. **114**: p. 2058–2075.
3. Bobbitt, J.M., *Oxoammonium Salts. 6. 4-Acetylamino-2,2,6,6-tetramethylpiperidine-1-oxoammonium Perchlorate: A Stable and Convenient Reagent for the Oxidation of Alcohols. Silica Gel Catalysis.* J. Org. Chem. , 1998. **63**: p. 9367-9374.
4. Zhu, X.Q., et al., *Thermodynamics and kinetics of the hydride-transfer cycles for 1-aryl-1,4-dihydronicotinamide and its 1,2-dihydroisomer.* Chem. Eur. J., 2003. **9**(16): p. 3937-45.
5. Zhu, X.Q., et al., *A classical but new kinetic equation for hydride transfer reactions.* Org Biomol Chem, 2013. **11**(36): p. 6071-89.

6. Abdullah, D.L.C.a.A.H., *Synthesis of l-Acyl-1,4-dihydropyridines via Copper Hydride Reduction of 1-Acylpyridinium Salts.* J. Org. Chem., 1984. **49**: p. 3392-3394.

SII. ^{1}H NMR and ^{13}C NMR of Some Representative Compounds

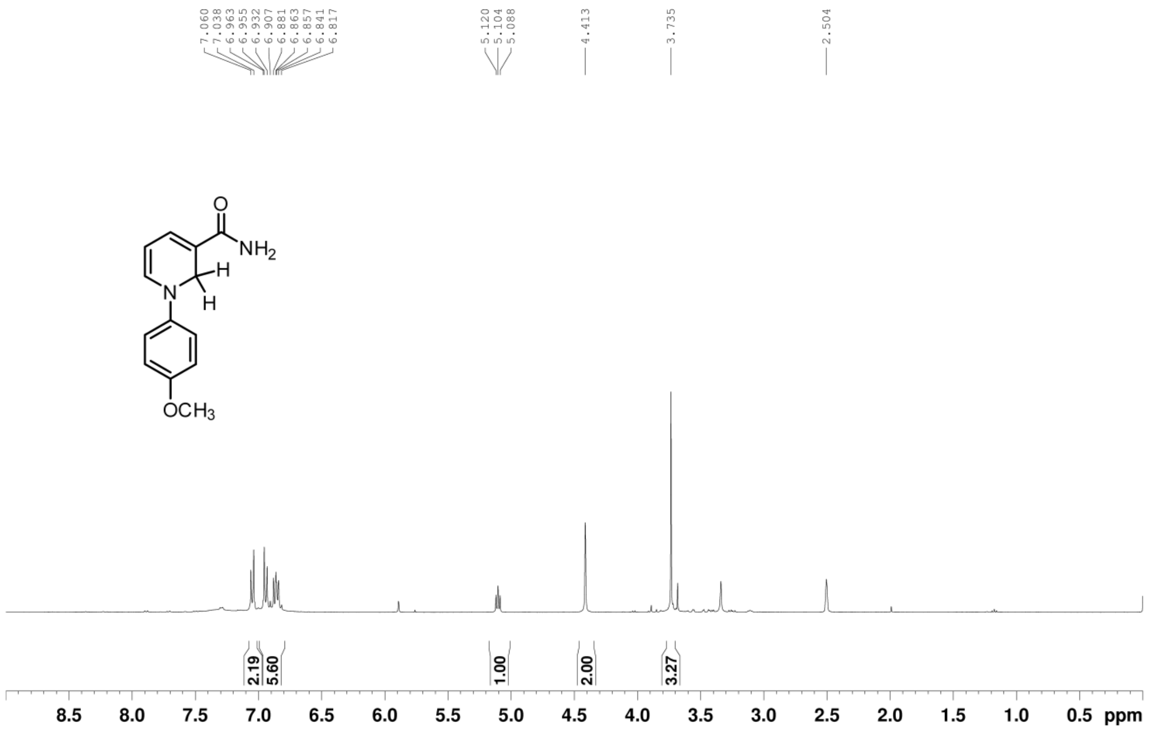

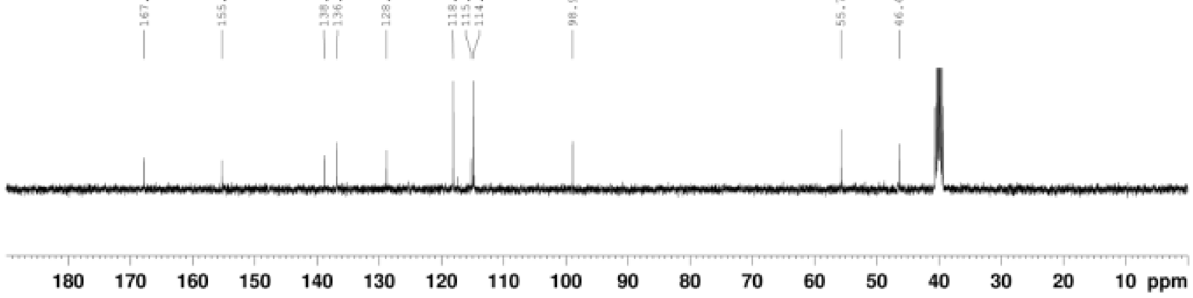

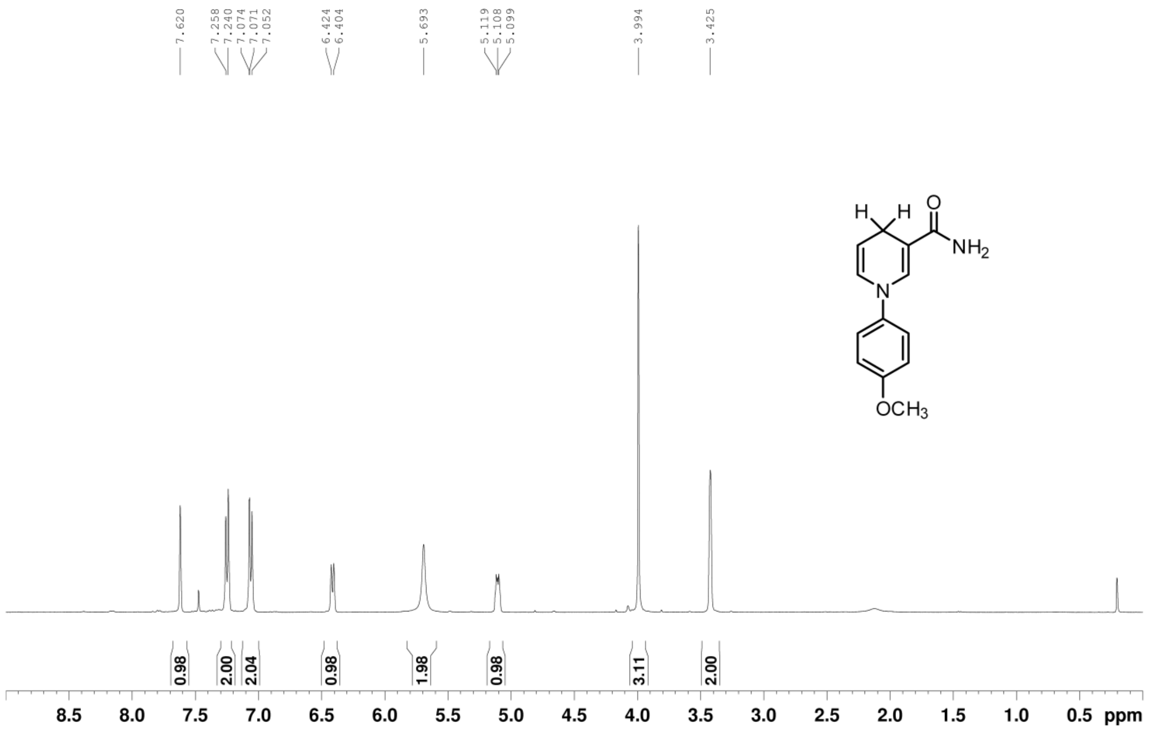
7.620
7.258
7.240
7.074
7.071
7.052
6.424
6.404
5.693
5.119
5.108
5.099
3.994
3.425
NH2
OCH3
0.98
2.00
2.04
0.98
1.98
0.98
3.11
2.00
ppm

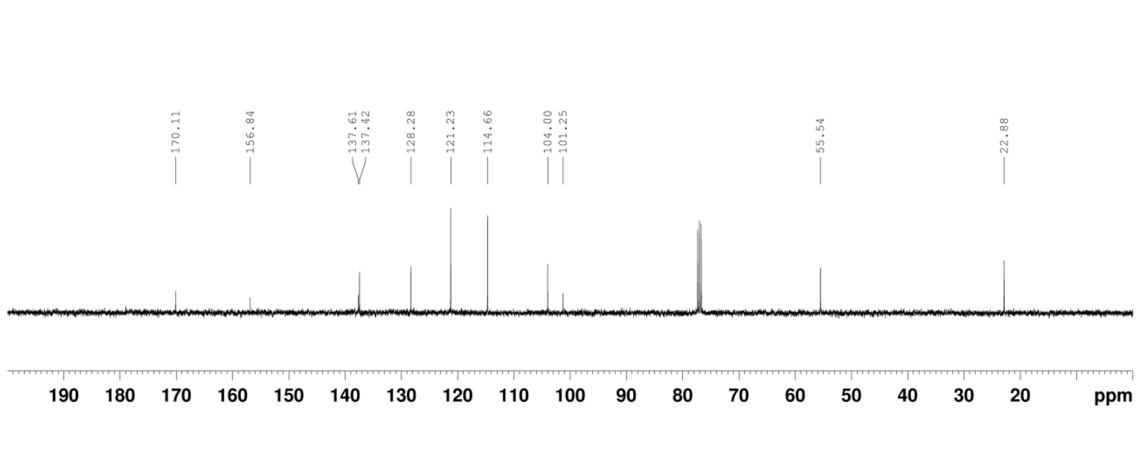
170.11
156.84
137.61
137.42
128.28
121.23
114.66
104.00
101.25
55.54
22.88
ppm

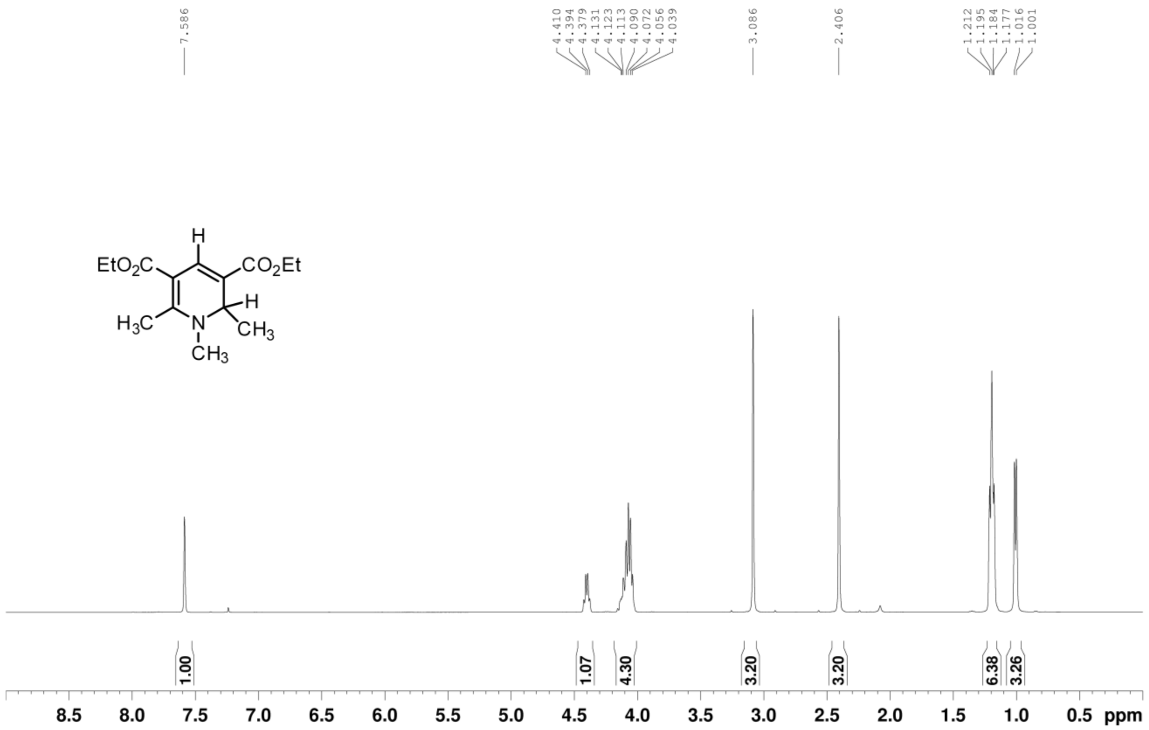
7.586
4.410
4.394
4.379
4.131
4.123
4.113
4.090
4.072
4.056
4.039
3.086
2.406
1.212
1.195
1.184
1.177
1.016
1.001
EtO2C
CO2Et
H
H
H3C
CH3
N
CH3
1.00
1.07
4.30
3.20
3.20
6.38
3.26
8.5 8.0 7.5 7.0 6.5 6.0 5.5 5.0 4.5 4.0 3.5 3.0 2.5 2.0 1.5 1.0 0.5 ppm

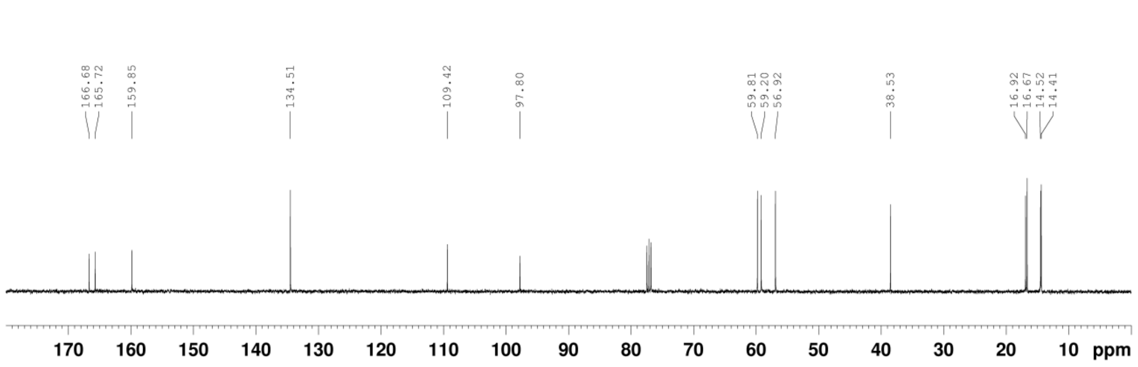
166.68
165.72
159.85
134.51
109.42
97.80
59.81
59.20
56.92
38.53
16.92
16.67
14.52
14.41
170 160 150 140 130 120 110 100 90 80 70 60 50 40 30 20 10 ppm

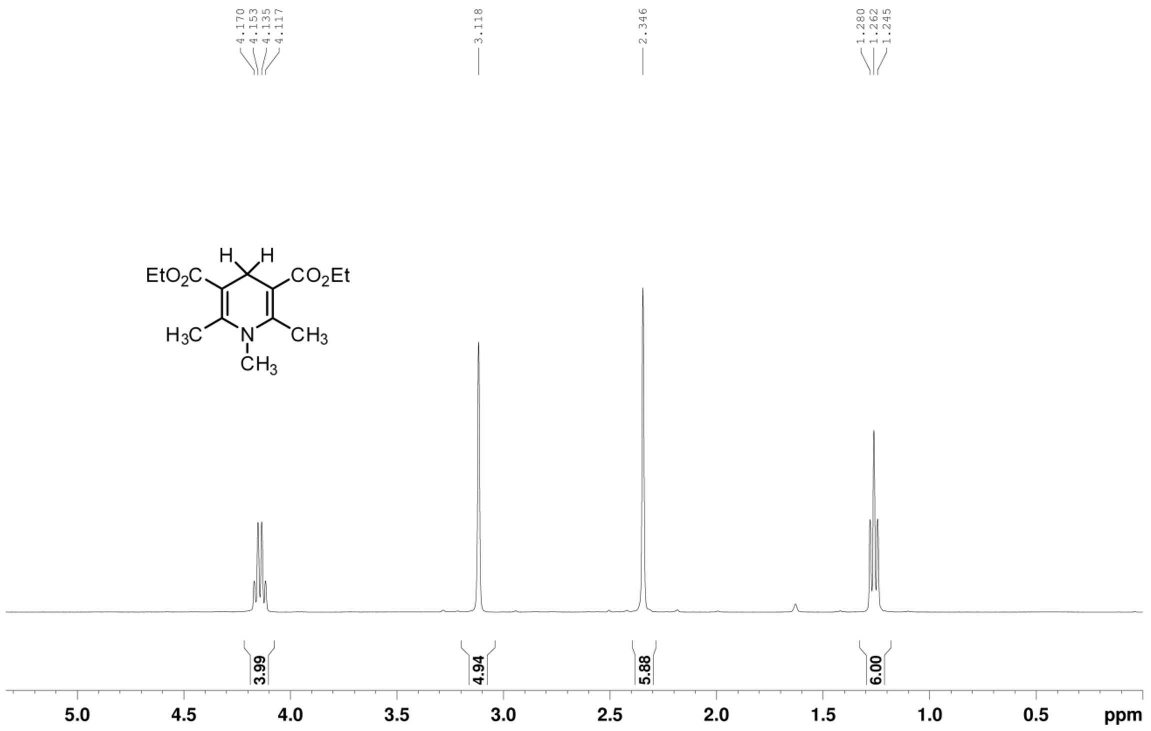
4.170
4.153
4.135
4.117
3.118
2.346
1.280
1.262
1.245
EtO_2C
CO_2Et
H
H
H_3C
N
CH_3
CH_3
3.99
4.94
5.88
6.00
5.0
4.5
4.0
3.5
3.0
2.5
2.0
1.5
1.0
0.5
ppm

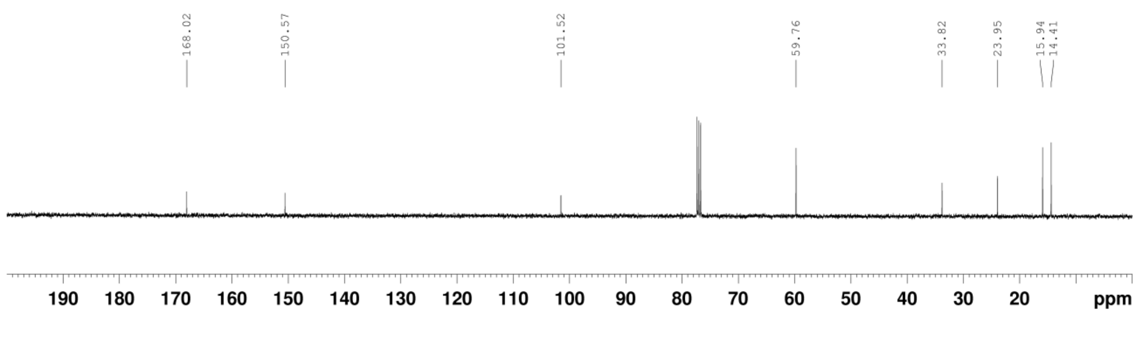
168.02
150.57
101.52
59.76
33.82
23.95
15.94
14.41
190
180
170
160
150
140
130
120
110
100
90
80
70
60
50
40
30
20
ppm

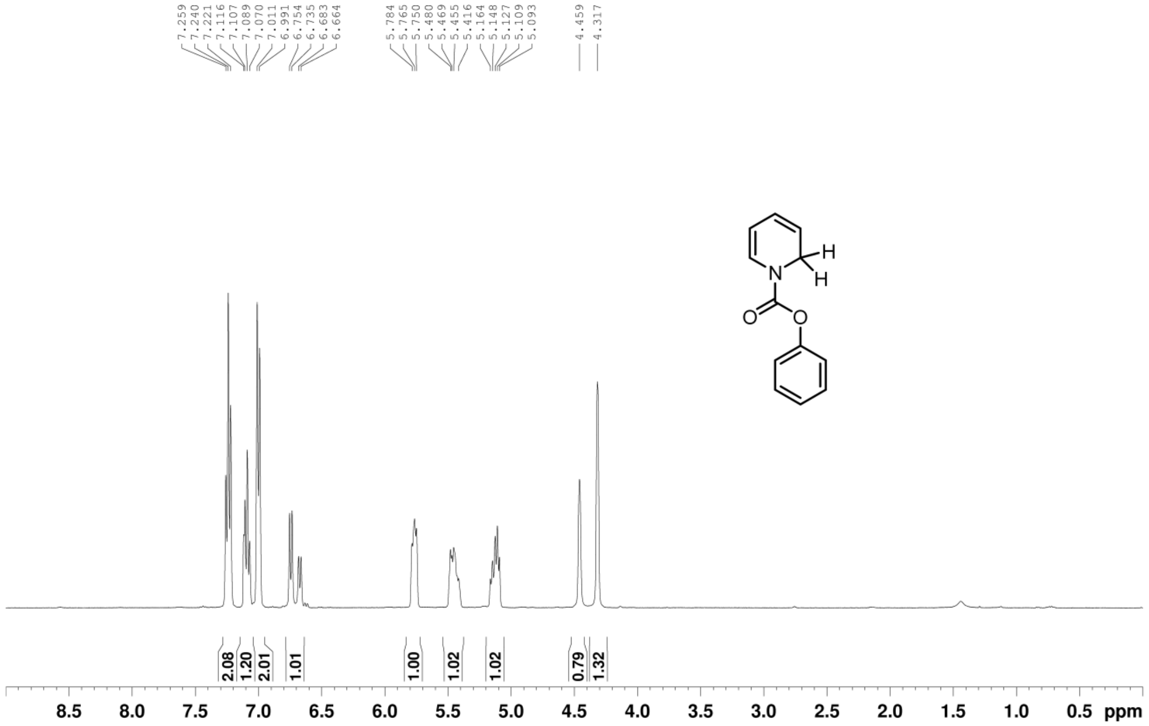
7.259 7.240 7.221 7.116 7.107 7.089 7.070 7.011 6.991 6.754 6.735 6.683 6.664
5.784 5.765 5.750 5.480 5.469 5.455 5.416 5.164 5.148 5.127 5.109 5.093
4.459 4.317
H
H
N
O
O
2.08 1.20 2.01 1.01 1.00 1.02 1.02 0.79 1.32
8.5 8.0 7.5 7.0 6.5 6.0 5.5 5.0 4.5 4.0 3.5 3.0 2.5 2.0 1.5 1.0 0.5 ppm

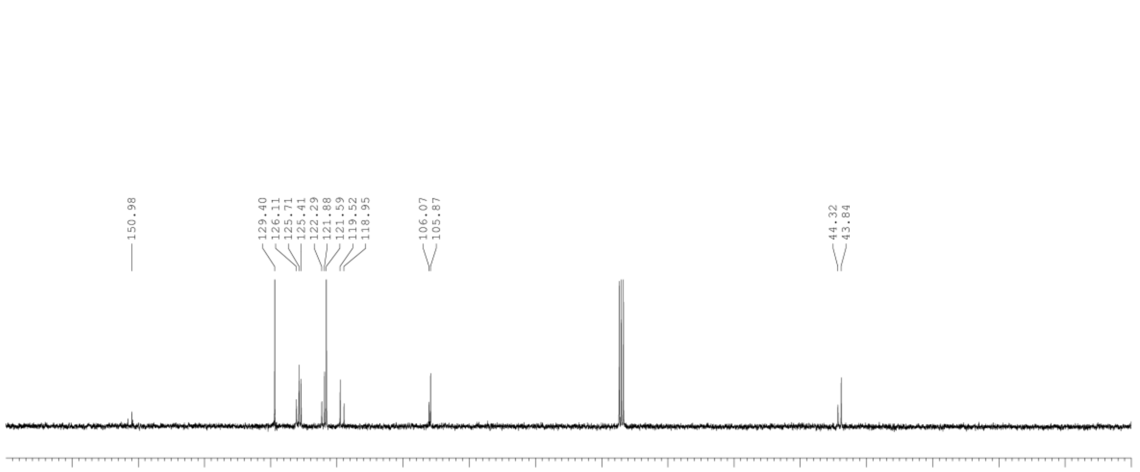
150.98
129.40 126.11 125.71 125.41 122.29 121.88 121.59 119.52 118.95
106.07 105.87
44.32 43.84
160 150 140 130 120 110 100 90 80 70 60 50 40 30 20 10 ppm

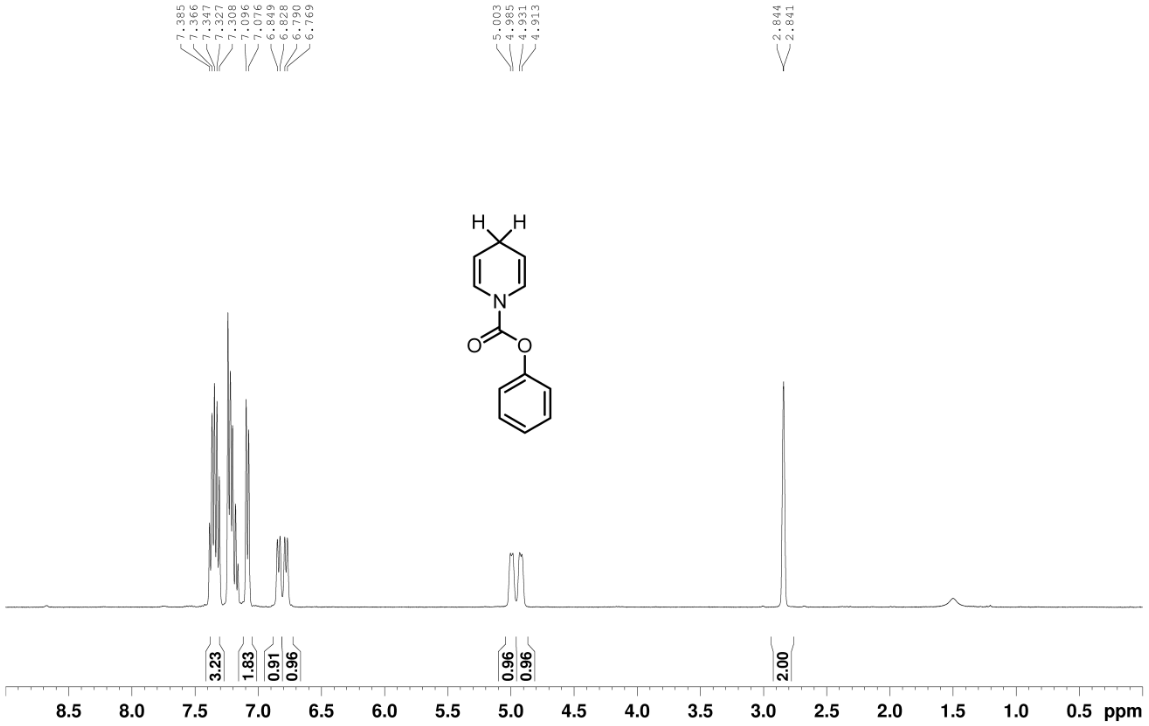
7.385
7.366
7.347
7.327
7.308
7.096
7.076
6.849
6.828
6.790
6.769
5.003
4.985
4.931
4.913
2.844
2.841
H
H
N
O
O
3.23
1.83
0.91
0.96
0.96
0.96
2.00
8.5 8.0 7.5 7.0 6.5 6.0 5.5 5.0 4.5 4.0 3.5 3.0 2.5 2.0 1.5 1.0 0.5 ppm

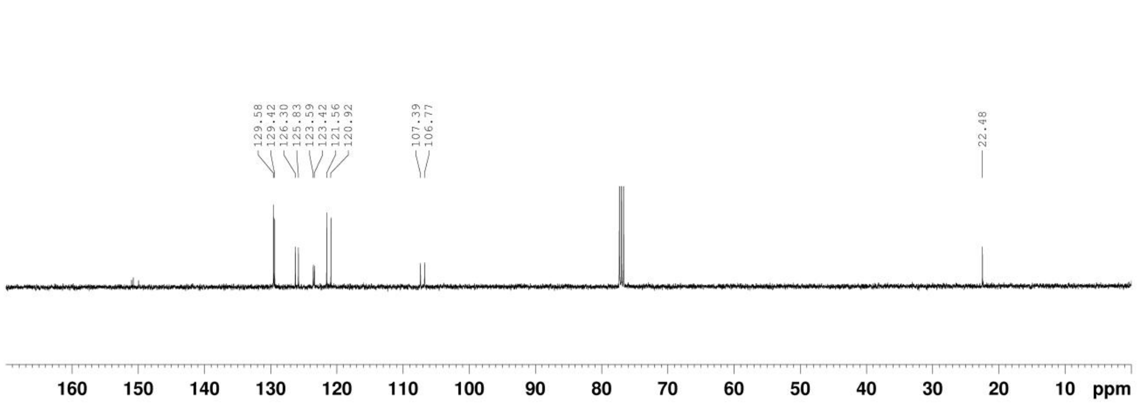
129.58
129.42
126.30
125.83
123.59
123.42
121.56
120.92
107.39
106.77
22.48
160 150 140 130 120 110 100 90 80 70 60 50 40 30 20 10 ppm
